# Time-Constrained Node Visit Planning for Collaborative UAV–WSN Distributed Applications

**DOI:** 10.3390/s22145298

**Published:** 2022-07-15

**Authors:** Andrea Augello, Salvatore Gaglio, Giuseppe Lo Re, Daniele Peri

**Affiliations:** 1 Department of Engineering, University of Palermo, Viale delle Scienze, Ed. 6, 90128 Palermo, Italy; andrea.augello01@unipa.it (A.A.); salvatore.gaglio@unipa.it (S.G.); giuseppe.lore@unipa.it (G.L.R.); 2 Institute for High Performance Computing and Networking (ICAR), National Research Council (CNR), Via Ugo La Malfa, 153, 90146 Palermo, Italy

**Keywords:** distributed computing, UAV, UAV–WSN integration, UAV path planning, UAV task scheduling, UAV-supported IoT systems for smart cities

## Abstract

Unmanned Aerial Vehicles (UAVs) are often studied as tools to perform data collection from Wireless Sensor Networks (WSNs). Path planning is a fundamental aspect of this endeavor. Works in the current literature assume that data are always ready to be retrieved when the UAV passes. This operational model is quite rigid and does not allow for the integration of the UAV as a computational object playing an active role in the network. In fact, the UAV could begin the computation on a first visit and retrieve the data later. Potentially, the UAV could orchestrate the distributed computation to improve its performance, change its parameters, and even upload new applications to the sensor network. In this paper, we analyze a scenario where a UAV plays an active role in the operation of multiple sensor networks by visiting different node clusters to initiate distributed computation and collect the final outcomes. The experimental results validate the effectiveness of the proposed method in optimizing total flight time, Average Age of Information, Average cluster computation end time, and Average data collection time compared to prevalent approaches to UAV path-planning that are adapted to the purpose.

## 1. Introduction

Wireless Sensor Networks (WSNs) are an important component of the emerging distributed computing paradigms of IoT, Smart Cities, and Ambient Intelligence [1]. The introduction of one or more Unmanned Aerial Vehicles (UAVs) can greatly enhance the abilities of a WSN. For instance, a UAV in a WSN can either act as a mobile sink or a probing node, perform maintenance operations, or bring connectivity to a network [2]. This can be especially useful in emergency scenarios where part of the network may be disrupted [3].

UAVs, however, may have limited computational capacity and severe energy constraints. Hence, the dispatch of a drone must be carefully planned, making the optimization of paths and drone operations a prominent issue [4].

Many scenarios where UAV path-planning is required to support a WSN have been analyzed in the recent literature with the aim of optimizing some problem-specific metrics. For example, UAV trajectory and communication scheduling has been the subject of optimizations aiming to guarantee the required quality of service in terms of average throughput for a UAV-assisted networking emergency application [3]. In that case, the proposed solution was based on classical optimization algorithms.

Some works focus on localizing Sensor Nodes (SNs) on the ground, with no prior knowledge of their position. For instance, a genetic algorithm was proposed to optimize the path length and flight time of a UAV while trying to perform localization of ground SNs using a range-free technique [5]. Other works use reinforcement learning to minimize the positioning error when trying to locate ground SNs with a UAV [6].

A popular problem that has been extensively studied is that of computation offloading. In this case, the UAVs collaborate with the WSN, acting as mobile edge servers for the SNs.

This task-offloading problem has been tackled by trying to minimize the overall energy consumption of both the SNs, either static or non-static, on the ground and the UAV [7]. A single UAV is used to carry out computation tasks on behalf of fixed and mobile ground devices in another work [8]. Successive convex approximations are used to maximize computational efficiency in terms of the ratio between offloaded data and UAV energy consumption. The problem of computational efficiency maximization has been further extended to the multi-UAV case, also including the possibility of partial computation offloading [9].

The offloading idea has also been extended to the possibility of forwarding data to an edge server on the ground [10] instead of performing all computation on board the UAV. In the same scenario, the computation offloading policy has been determined through reinforcement learning with improved results with respect to the strategy of sticking to a single policy [11].

A use case that raises great interest is that of employing UAVs for data collection in WSNs [12]. The use of a UAV as a mobile sink node is one of the most commonly studied scenarios, entailing collaborations between UAVs and WSNs [13]. UAV-aided data collection not only increases the expected lifetime of the network but also reduces data processing and memory-related issues in the network [14].

A UAV can be used to extend the network lifetime by wirelessly charging the SNs when performing data collection [15].

The energy constraint of the UAV can also be a limiting factor in joint UAV-WSN operations. The authors of [16] use Q-learning to determine a flying route that enables data to be collected from remote SNs, while simultaneously maintaining acceptable energy levels. The UAV energy constraints are mitigated by assuming the presence of wireless UAV charging stations.

Data collection can also be subject to timing constraints: SNs have limited memory and may drop data that are not collected by the UAV in a timely manner. Node visit scheduling algorithms that outperform a naive, greedy flight strategy for this scenario have been proposed [17] for this purpose.

Even if not dropped by the SNs, data can quickly become outdated. The impact of the Age of Information (AoI) on UAV path-planning for data collection in a sensor network is investigated by the authors of [18]. AoI is defined by the authors as the time that elapses from data collection to the UAV landing. Minimizing the AoI in this scenario is shown to be equivalent to solving a Traveling Salesman Problem (TSP). AoI is then minimized using both dynamic programming and a genetic algorithm with up to 14 SNs, outperforming the greedy approach.

The positioning of the SNs can influence the algorithms used for path planning. If the SNs lie on a line, the visiting order is fixed and the total flight time can be minimized by modulating the UAV flying speed to collect data on the fly [19].

Data collection applications exploiting the positional information of SNs are further explored in [20]. In this case, the UAV can collect data from multiple physically close SNs from the same hovering position. After hovering positions are determined, SNs are assigned to one of these and the best path that visits all the waypoints is found by solving a TSP with energy consumption constraints.

If nodes are organized in clusters, the UAV only needs to retrieve data from each sink node, also referred to as Cluster Head (CH), instead of collecting data from multiple nearby SNs. Deep reinforcement learning has been used to design routes for the UAV to collect data from the CHs, outperforming Nearest Neighbor and genetic algorithms [21].

To the best of our knowledge, all UAV-aided data collection scenarios in the current literature assume that the data required by the UAV are either available on the nodes or computed by the nodes in a negligible time with respect to that of a visit to all the nodes by the UAV. This assumption may not necessarily hold true and limits the possible scope of the integration of UAVs and sensor networks.

In more sophisticated applications, such as maintenance, configuration, and testing operations, a UAV may send the code of a distributed algorithm whose execution could last several minutes to a network. The UAV-carried distributed algorithm could even self-propagate through a network, before eventually being delivered to every cluster. Making a deployed WSN execute distributed applications through the dynamic exchange of high-level symbolic code was demonstrated to be effective even when SNs are resource-constrained [22]. These distributed applications may be useful when assessing the state of the nodes [23] and for maintenance operations in networks that are otherwise not serviceable. The symbolic distributed programming approach was also proven to be able to support the verification of both hardware proper functioning [24] and the correctness of distributed applications on deployed WSN [25].

The use of UAV to control a WSN symbolic distributed application would be relevant to multiple real-world use-cases. For instance, in an emergency scenario, a UAV could be used to inject a network discovery application [23] in a WSN to assess which nodes are still functioning. Regarding maintenance operations, as the UAV substitutes malfunctioning nodes [26], it may also verify the soundness of the new network configuration, in terms of both connectivity [27] and high-level protocol execution [28]. Finally, even in the case of regular sensing operations, sending a UAV to start the sampling operations only when they are needed and retrieve the data when they are ready could reduce the energy consumption of the network.

In this work, we consider a scenario in which a single UAV initiates the execution of distributed applications and collects the computation results in a WSN comprising several disjoint clusters with static CHs and propose a method for effective flight planning.

The rest of the paper is structured as follows. Section 2 details the proposed scenario and introduces a mathematical model to deal with it. Section 3 describes our approach. Section 4 presents the experimental results. Finally, Section 5 reports our conclusions and directions for future works.

## 2. Problem Statement and Model Formulation

**Figure 1:**
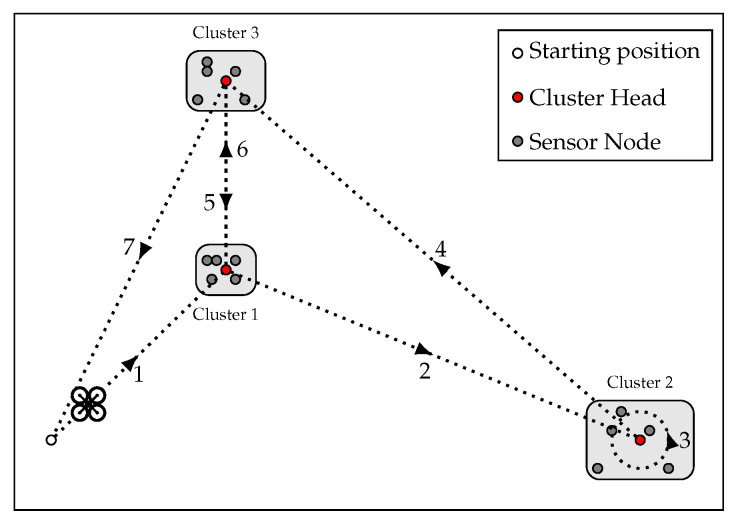
Example scenario. The UAV either visits each CH twice or hovers (circle around the CH of cluster 2) over a CH until the computation ends. The number near each arrow indicates the visit order.

An instance of the scenario considered in this work is shown in Figure 1. The UAV departs from a fixed starting position and reaches every CH to initiate the execution of a distributed application. Later, the UAV collects the resulting data from the same CHs. While the application is running, the UAV can hover in place, waiting for the computation to be completed, or move to other clusters. Both displacement and hovering have a cost in terms of either the time required to reach the destination or time spent waiting in place. CHs can be visited in any order, and data can be collected from some clusters before others have been visited.

Let $W=\{w_{1},\ldots ,w_{n}\}$ be the set of waypoints corresponding to the CHs, and $W_{0}=\left\{w_{0}\right\}\cup W$ the set including the UAV starting position. The UAV needs to visit each CH twice: the UAV goes to $w_{i}$ once at $t_{i}$ to start the execution of a distributed application, and a second time at $t_{i^{\prime }}$ to collect the computation results. While the SNs of a cluster execute their application, the UAV is free to visit other CHs to collect data or start another execution.

The execution time for the application, $\tau_{i}\;\forall w_{i}\in W$, may depend on the characteristics of the cluster, e.g, the topology or the number of nodes in the cluster. However, the execution time is assumed to be deterministic and known.

**Figure 2:**
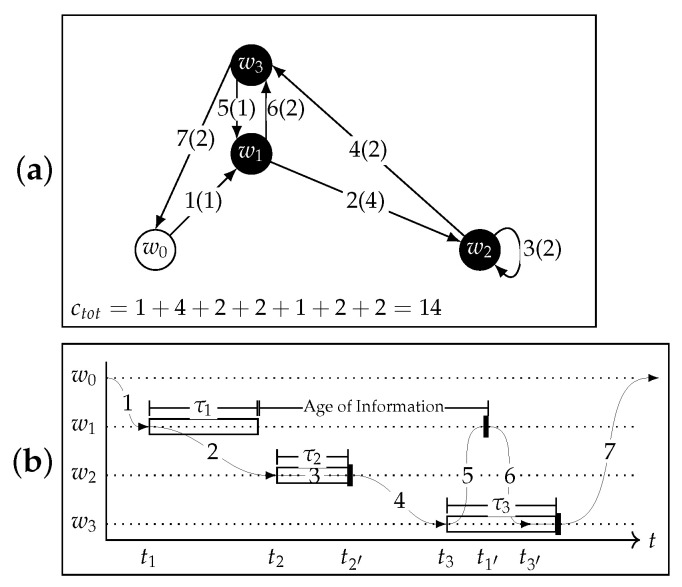
Path shown in terms of both the visit path through waypoints $\left[w_{0},w_{1},w_{2},w_{2},w_{3},w_{1},w_{3},w_{0}\right]$ (**a**) and the corresponding execution of processes on the nodes (**b**). The visit sequence has two consecutive $w_{2}$ because the UAV hovers there until the application execution in cluster 2 is over. The cost of each edge is shown in the round brackets next to the visit-order number. The empty rectangles correspond to the execution of the distributed computation, each associated with the respective execution time $\tau_{i}$; the filled rectangles represent data collection operations.

As a practical example, the model in Figure 1 with its three CHs is depicted with more details in Figure 2. Figure 2a represents a possible path, touching all the waypoints. The visiting order is shown for each of them, along with the arc cost enclosed in round brackets.

The UAV takes off at $w_{0}$, then heads toward $w_{1}$, where the execution of a distributed application is initiated. After this, the UAV reaches $w_{2}$, starts the application, hovers in place until completion, and finally collects some data. The next visited cluster to begin computation corresponds to $w_{3}$. Meanwhile, the data at $w_{1}$ are ready; the UAV collects them and heads back to $w_{3}$, where it has to wait for the computation to end (as shown in Figure 2b). Then, data collection is finished and the UAV can return to $w_{0}$.

In Figure 2b, the same sequence of actions is represented in terms of the process execution on the clusters. The empty rectangles correspond to the execution of a distributed computation process on a cluster; the filled rectangles represent data collection operations. In the distributed computation execution, the CH at waypoint $w_{i}$ starts a process at $t_{i}$ that lasts for the execution time $\tau_{i}$. Then, at time $t_{i}^{\prime }$, the UAV collects the output data from the CH at $w_{i}$ For cluster 1, once the data are ready, some time will elapse before data are collected by the UAV. This time interval is labeled as the AoI in the figure.

Given a pair of waypoints $w_{i},w_{j}\in W_{0}$ going through the edge e(i,j), has a time cost that is equal to the flight time f(i,j), proportional to the physical distance between $w_{i}$ and $w_{j}$ and inversely proportional to the UAV flying speed. Of course, $f\left(i,i\right)=0\;\forall i\in W_{0}$.

The cost $c_{ij}\left(t\right)$ of visiting the waypoint $w_{j}$, departing from $w_{i}$ at time *t*, is then given by Equation (1). This cost does not depend on whether the starting node was visited for the first or second time. The cost formulation considers that, if $w_{j}$ is reached before it completed its computation, the UAV will need to wait before departing for the next node.

(1) $$c_{ij}\left(t\right)=\left\{\begin{array}{cc} f(i,j) & \mathrm{visit}\;1\\ \tau_{i} & i=j\\ \max\{f\left(i,j\right),t_{j}+\tau_{j}-t\} & \mathrm{visit}\;2 \end{array}\right.$$

The objective of an algorithm that aims to find the optimal path is then to find a sequence of waypoints $u=[w_{0},\ldots ,w_{i},w_{j},\ldots ,w_{0}]$ that starts at $w_{0}$, includes each waypoint in *W* twice, and ends at $w_{0}$, such that the total cost of the path is minimized.

## 3. Proposed Approach

**Figure 3:**
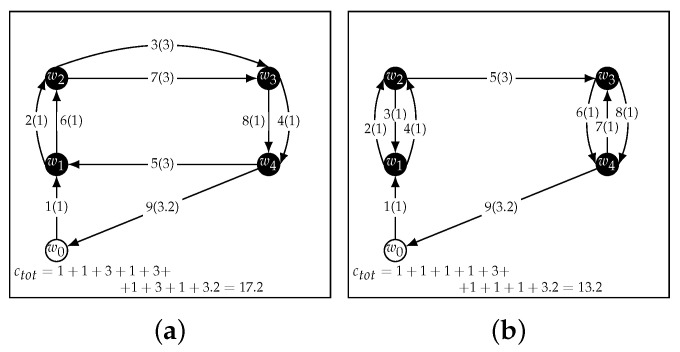
Comparison of the Double Round (**a**) strategy and a greedy strategy (**b**). The latter produced a better path.

A naive solution to this problem would be to find an optimal Hamiltonian cycle [29], which is the shortest path that visits every node exactly once, for $W_{0}$ and another one for *W*. Then, to visit each CH twice, the UAV would have to go through the first cycle up to the last waypoint, then the second cycle, to finally return to the start position $w_{0}$. This strategy is shown in Figure 3a. From now on this, strategy will be referred to as Double Round.

Although at a first glance this may seem a reasonable strategy, it explores a limited subset of the 2n! possible paths and may not always find the optimal route. For instance, Figure 3b shows how a path found through a greedy algorithm can be shorter than the one found with Double Round.

**Figure 4:**
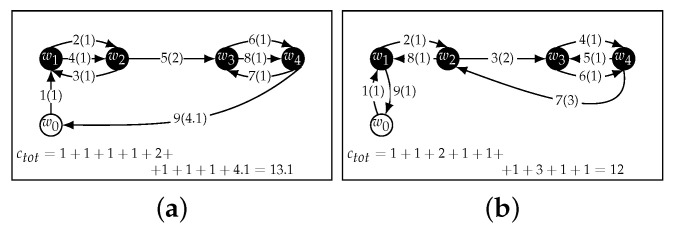
A greedy strategy (**a**) can lead to less than optimal paths (**b**).

Greedy strategies, however, are known to sometimes yield poor solutions for this class of routing problems [30]. In Figure 4, a greedy approach is shown to find a suboptimal path (a) even though a shorter path exists (b).

**Figure 5:**
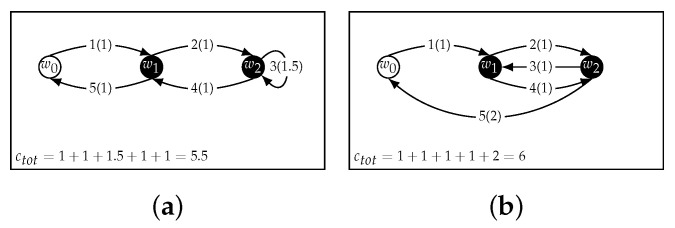
Permitting the UAV to hover on a waypoint (**a**) may lead to shorter paths than visiting other clusters and then going back (**b**).

Another factor to consider is that, depending on the execution time of the distributed application on a given cluster, hovering in place until completion (Figure 5a) may be preferable to visiting other clusters and then going back (Figure 5b). This possibility is intrinsic to the approach we present in the following.

**Figure 6:**
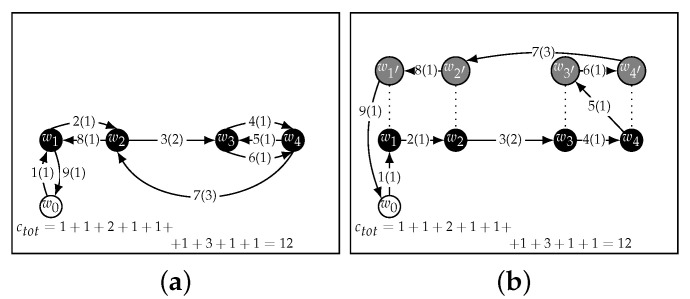
The same path as in Figure 4b is shown both as a path that visits each node twice in the original graph (**a**) and as a Hamiltonian cycle in a graph with duplicate nodes (**b**). The duplicate nodes are all visited after the original nodes.

To find an optimal route to start the application execution and collect data for *n* clusters, we turn this problem into a TSP over 2n+1 waypoints. Instead of finding a route that involves each waypoint in *W* twice, we introduce a second set of waypoints $W^{\prime }=\left\{w_{1^{\prime }},\ldots ,w_{n^{\prime }}\right\}$. These waypoints are in the same physical locations as the waypoints in *W*, so that $f\left(w_{i},w_{i^{\prime }}\right)=0\;\forall w_{i}\in W,w_{i^{\prime }}\in W^{\prime }$. The task of finding the optimal path then turns into finding the Hamiltonian cycle over $W_{0}\cup W^{\prime }=\left\{w_{0},w_{1},\ldots ,w_{n},w_{1^{\prime }},\ldots ,w_{n^{\prime }}\right\}$ (Figure 6) with minimal costs.

When the UAV reaches a CH for the first time, the visit is attributed to the waypoint in *W*. The corresponding waypoint in $W^{\prime }$ is considered to be the one visited second or after hovering in place. The arc costs in this alternative problem formulation are given by Equation (2).

(2) $$c_{ij}\left(t\right)=\left\{\begin{array}{cc} f(i,j) & w_{j}\in W_{0}\\ \max\{f\left(i,j\right),t_{j}+\tau_{j}-t\} & w_{j}\in W^{\prime } \end{array}\right.$$

Note that if $w_{j}=w_{i^{\prime }}$ then f(i,j)=0 and $t=t_{j}$, we have that $c_{ij}=c_{ii^{\prime }}=\tau_{i}$ and $t_{i^{\prime }}=t_{i}+\tau_{i}$.

As we have shown, this problem is equivalent to a TSP instance, so it is also NP-hard. Moreover, since the search space has 2n! possible solutions, trying to find an exact one is more demanding than finding one among the n! possible solutions of a regular TSP. Thus, we did not consider exhaustive searches. It should be noted that, while beyond the scope of this work, the proposed model can easily be extended to include an arbitrary number of visits to the same clusters by including more sets of duplicate nodes.

**Figure 7:**
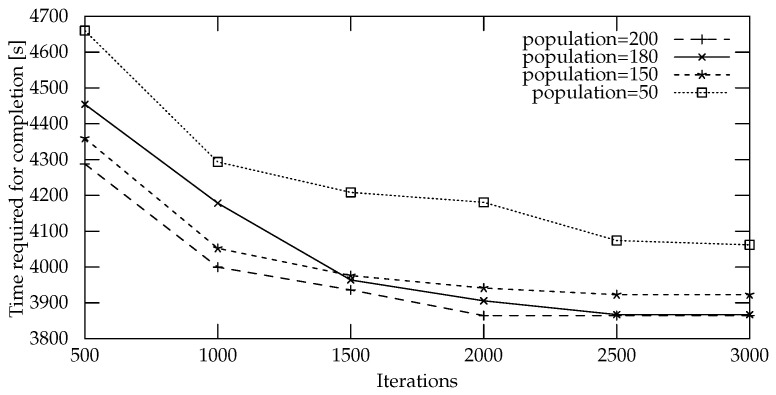
Preliminary results for 100 waypoints at increasing population sizes and numbers of iterations used for parameter fine-tuning.

Swarm intelligence algorithms are a popular approach to UAV path planning [31,32]. We used the Bees Algorithm (BA) [33]. This algorithm performs both local and global searches through exploitation and exploration strategies: the algorithm starts with a population of scout bees that explore random locations in the search space, and the neighborhood of the best locations is explored by forager bees, while the scouts that found the worst locations abandon them to search for other random locations. This process is iterated until a stop condition is met. In our implementation, we used a population of 180 bees. As suggested by the literature [34], 20% of the total population were scouts. The stop condition for the algorithm was chosen as a fixed limit of 2500 iterations. The population size and number of iterations were chosen as the result of some preliminary experiments (Figure 7) that showed no added benefits to using larger populations or more iterations.

The computational cost of our BA implementation is proportional to both the population size and the number of iterations. In the remainder of the paper, our proposed solution to the problem using BA over the set of 2n+1 waypoints will be referred to as the $BA_{2n+1}$ strategy. As shown in Section 4, randomly choosing the starting location in the search space led to an overall good performance of the strategy. However, it did not always provide the best solutions. We thus decided to integrate the strategy with more options for the initialization step, as shown in Section 4.1, further improving the results.

## 4. Experimental Evaluation

**Figure 8:**
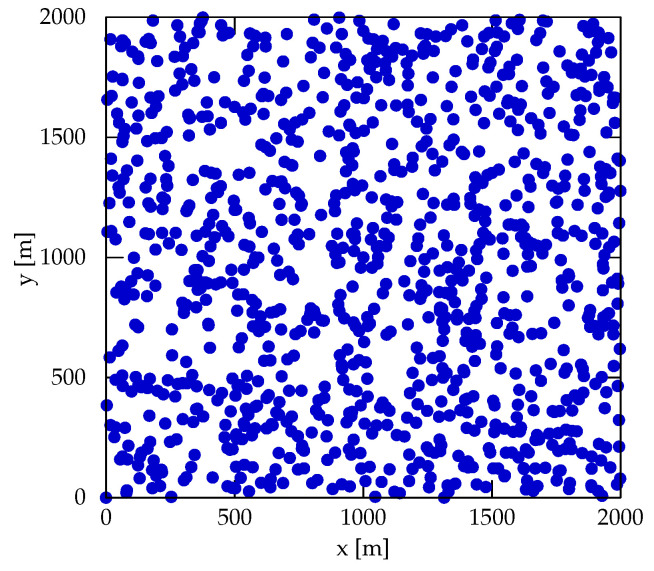
Overall coverage of the area of operation (2000 m × 2000 m) by the different experimental arrangements of 100 clusters.

In order to assess the feasibility of the BA strategy, we performed extensive numerical simulations using Python (3.8.10) and NumPy (1.22.0). The UAV is assumed to have a constant flying speed of 11 m/s through the whole simulation. This parameter choice is in accordance with other works in the literature [6]. We decided to use arrangements of 5, 10, 20, 50, and 100 clusters, scattered into a 2000 m × 2000 m area sampling from a uniform random distribution for CH coordinates (Figure 8). The number of clusters and area size were chosen according to reports in the literature for UAV sensing operations [35].

For each number of clusters, 10 arrangements were generated using different random seeds. For each arrangement, the simulations were performed with three possible UAV starting positions: the center of the area, a corner, and a random position within the area. Five choices for the number of clusters, ten arrangements per each choice of number of clusters, and three possible UAV starting positions resulted in 150 test configurations.

The application execution time for each cluster was randomly assigned from a 2–5 min range by uniform sampling. Given the typical flight duration abilities of a UAV, which is usually measurable in a few tens of minutes [35], a longer application execution time would not be realistic in the context of a single flight mission. The selected range is also comparable with the time the UAV takes to move from one CH to another.

We compared our $BA_{2n+1}$ strategy to the following ones:

1. The *Double Round strategy*, previously described in Section 3, which we implemented using the Google OR-Tools solver for routing problems [36]. This solver has been shown as able to match and sometimes outperform state-of-the-art reinforcement learning approaches for large problem spaces [37].
2. The *Single Round with Wait* strategy, which consists of finding the shortest path that visits each CH once and waiting in place until data collection before moving to the next waypoint. This strategy was implemented using the Google OR-Tools solver.
3. The *Greedy* nearest neighbor search strategy, as shown in Figure 3b and Figure 4a. This strategy has a time complexity of $O(n^{2})$, with *n* being the number of clusters.

We also performed comparisons with two more strategies that we derived from our 2n+1-waypoint formulation, but adopting other optimization methods:

4. The $OR_{2n+1}$ strategy using the OR-Tools solver.
5. The $GA_{2n+1}$ strategy based on the genetic algorithm described in [38], which is one of the most popular algorithms for UAV path-planning [39]. A population of size 200 was used, with 2-opt mutation [40], roulette wheel selection [41], and two-point crossover with repair. Our implementation of the genetic algorithm has a computational cost that is proportional to the population size and the number of generations.

In all the considered strategies, path-planning is performed offline by a resource-rich device. The UAV simply receives the computed, ordered list of waypoints to visits with no additional burden on its computational abilities.

**Figure 9:**
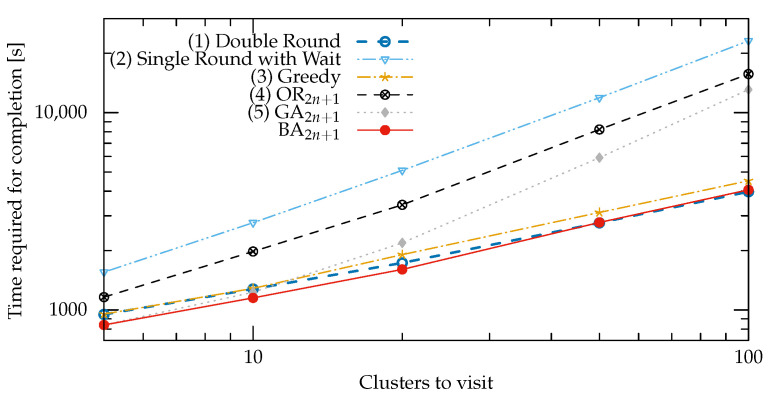
Average time required for mission completion versus number of nodes clusters to be visited. All scales are logarithmic.

The experimental results for the selected strategies at an increasing number of clusters are summarized in Figure 9.

Waiting for application execution at each cluster (Strategy 2) gave the worst results for every network size, confirming the unfeasibility of directly extending traditional approaches to the analyzed scenario.

Trying to solve our formulation of the optimization problem as with strategy 4 also led to poor performances.

The genetic algorithm started out with good results for a few clusters, in accordance with the current literature [18], but the quality of the obtained solutions quickly degraded as the number of clusters increased.

The three top-performing strategies were Double Round (strategy 1), Greedy (strategy 3), and BA, so we focused on these in the rest of the section.

**Figure 10:**
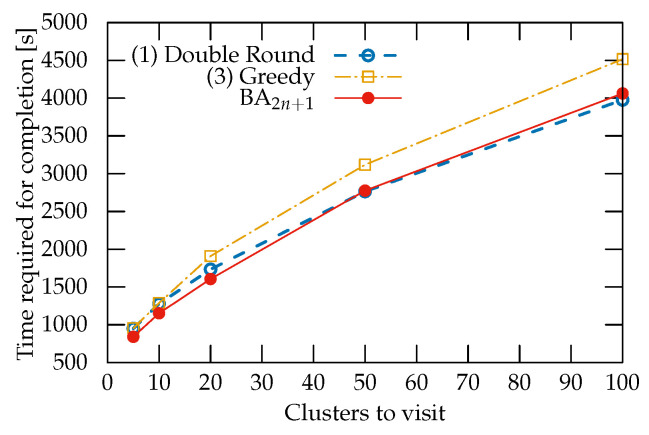
Average time required for mission completion versus number of node clusters to be visited. Only the three top strategies are shown.

BA outperformed all the other strategies when used with fewer than 100 clusters. With 100 clusters, Double Round produced 1.97% faster paths compared to BA (Figure 10). This drop in performance is attributed to the different rates at which the solution space increases for the two strategies: O(n!) for the former and O(2n!) for the latter.

### 4.1. Search Algorithm Initialization

**Figure 11:**
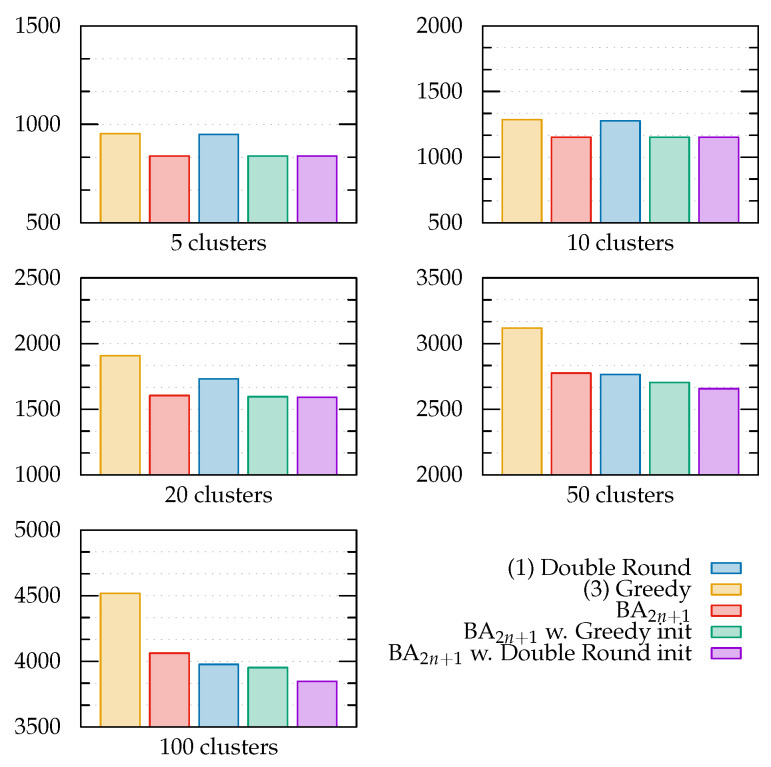
Performance of the selected search strategies with increasing number of clusters. The performance metric is still the time required for mission completion, measured in seconds.

Based on the first experimental result, we decided to focus on the neighborhood search part of the BA strategy in a promising region of the search space by splitting the initialization of the algorithm into two steps. In the first step, a good initial solution was found through either a Greedy search or Double Round. In the second step, the found solution was used as the starting state of one of the scout bees, while the others were randomly initialized as usual. Then, the BA algorithm ran unmodified with the same parameter choice described in Section 3. The performances of these primed BAs compared to the other strategies are shown in Figure 11.

BA improved the performance of the path found by the Greedy strategy by up to 12.64%, outperforming the Double Round strategy even with 100 clusters, albeit by a small margin (0.60% less total time). Priming BA with the Double Round path led to even better results, reaching a 3.22% improvement compared to Double Round, and was overall 5.27% less than unprimed BA with 100 clusters.

This version of BA, referred to as *$BA_{2n+1}$ + Double Round* in the following, was considered for the rest of the assessments, which target other desired properties of the interaction between UAVs and SN nodes.

**Figure 12:**
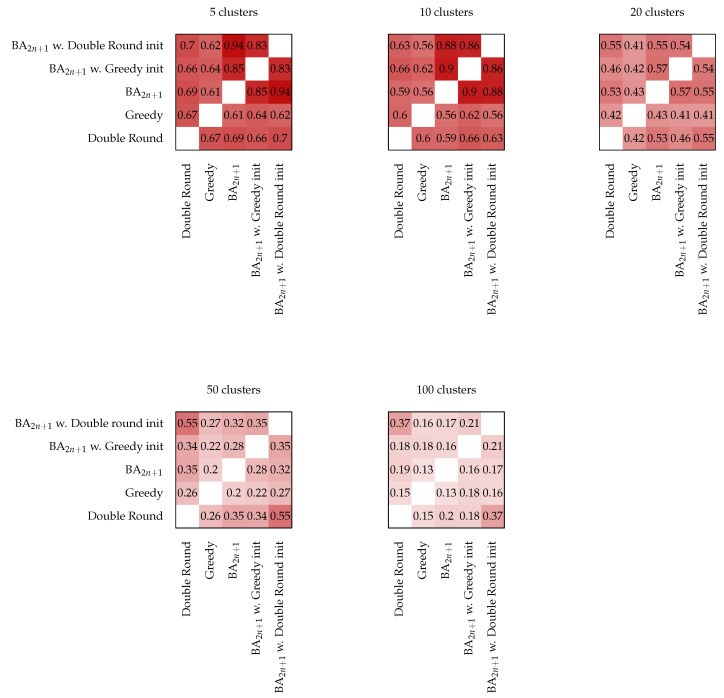
Pairwise path similarity based on their edit distance. As the number of clusters grows, the similarity between the paths found by the initialized BA and by the algorithm used for initialization decreases.

To better assess how much of the path used for initialization is maintained in the final path, we computed the average edit distance between the paths found by pairs of different strategies. Then, we used the values to obtain a similarity score (Figure 12). We used the Levenshtein distance (lev) [42] normalized in the [0,1] interval by dividing this by the total number of nodes in the paths (1−lev/n). This similarity corresponds to the fraction of the subsequences that match in the two paths. For up to 50 clusters, the paths found by BA, primed with Greedy initialization, were consistently more similar to the paths found by the unprimed BA than the one found by Greedy. The same is true for the Double Round priming with up to 20 clusters.

Overall, for few clusters, a high similarity can be noticed between all the paths, as the smaller solution space allows for all the techniques to converge to similar solutions in terms of both the performance and the structure of the found paths. On the other hand, the differences in performance with more clusters are obtained through BA by extensively editing the path used for initialization. As the number of clusters increased, the similarity to the path used for priming decreased, indicating that the search algorithm does not simply perform a local optimization but can conduct extensive explorations of the solution space.

**Figure 13:**
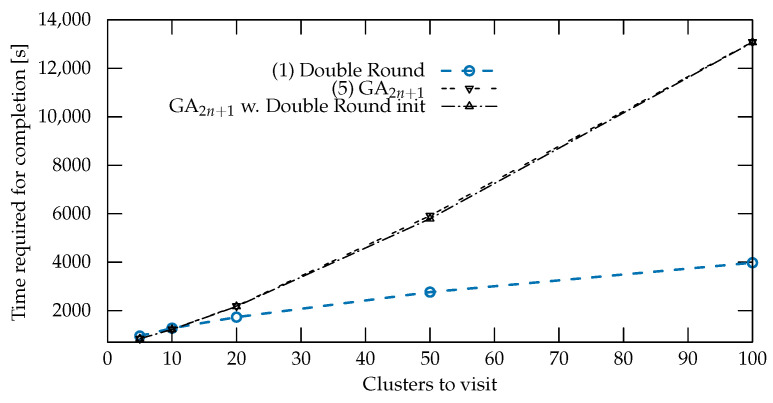
Average time required for mission completion versus number of nodes clusters to be visited. The improvement in the performance of the genetic algorithm given by the priming is negligible.

To further verify the suitability of the chosen search algorithm in light of this optimization of an initial path, we performed the same priming operation for GA (strategy 5). The results of this evaluation are summarized in Figure 13.

In this set of experiments, the priming operation had little impact on the performance of the GA, which improved only marginally and remained significantly worse than the performance of the Double Round. These experiments showed that optimizing the initialization does not necessarily improve the quality of the solutions, as is the case with BA.

### 4.2. Optimization of Other Time-Related Metrics

Energy-intensive activities such as exchanging a large amount of messages, as in CH, can shorten the lifetime of sensor network nodes. In paradigms such as transient computing, nodes may only be active for short periods. Hence, in addition to minimizing the overall duration of the path, in some circumstances, other metrics (Table 1) may be more important:

- Average AoI: once the computation is over, it is desirable to retrieve results as soon as possible, thus minimizing the chances of a node browning out while waiting until data are transmitted to the UAV. Moreover, in some scenarios, such as emergencies and industrial control, information may fast become obsolete as time passes. In Section 2, we reported the definition of AoI as the time elapsed from the moment data are available to the instant they are retrieved from the CH (see also Figure 2).
- Average cluster computation end time: for some operations, e.g., calibration, retrieving the end result may not be as important as minimizing the average time from mission start taken by a cluster to complete its computation.
- Average data collection time: in the event of having to abort a mission midway, retrieving as much data as possible early on minimizes the damage of not completing the mission.

**Figure 14:**
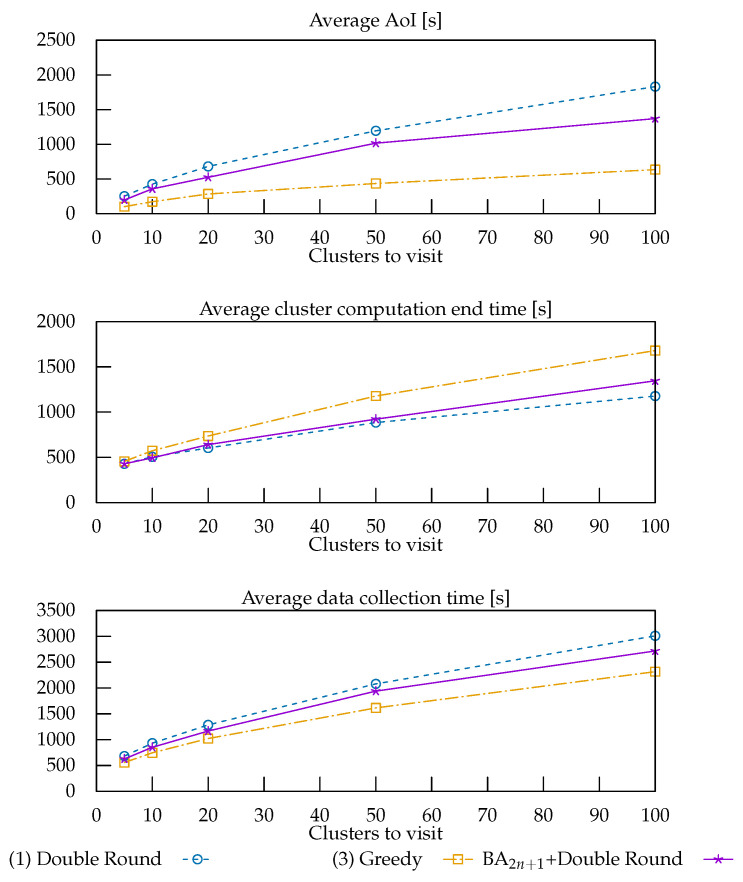
Performance of the strategies that produced the fastest routes with respect to other metrics.

The results of the evaluations of the solutions provided by the $BA_{2n+1}$ + Double Round, the Double Round, and the Greedy strategies to solve the problem of minimizing the total time with respect to the additional metrics are reported in Figure 14.

As expected, since its first round includes all the clusters, the Double Round strategy minimized the time required for nodes to end their computations. The Greedy strategy, however, outperformed the other two with respect to the other metrics, as privileging nearby clusters brought down the averages, albeit at the cost of a higher upper bound. $BA_{2n+1}$ + Double Round mitigated the shortcomings of the Double Round strategy, maintaining a similar performance for the average computation end.

**Figure 15:**
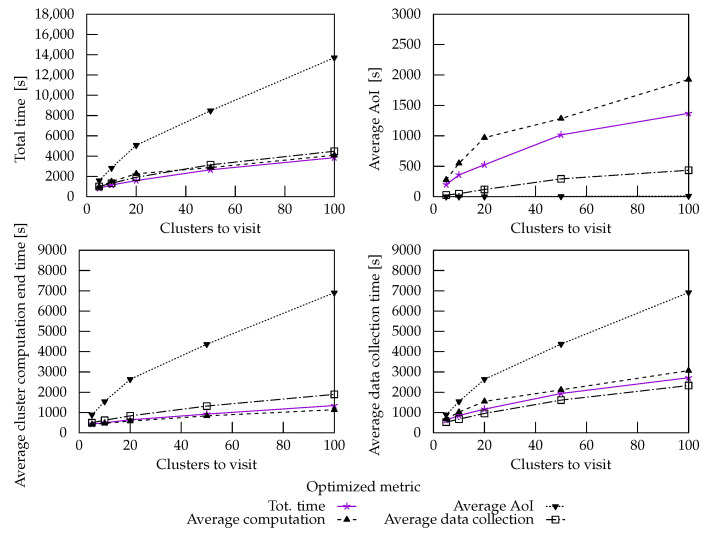
Performance of BA${}_{2n+1}$ with Double Round initialization at an increasing number of clusters on all the selected metrics, depending on the optimized metric.

Then, we used the $BA_{2n+1}$ + Double Round to explicitly minimize the other metrics and to better assess their relationship. The results of these experiments are reported in Figure 15.

Minimizing the average cluster computation end time produced paths, which visited many clusters first so that they started their computation as early as possible. Subsequently, the strategy scheduled the second visits for data collection and the remaining first visits. Overall, the emerging behavior was similar to that of the Double Round strategy.

Unsurprisingly, minimization of the average data collection time led to performances that were not dissimilar from those of total mission time minimization. This strategy also showed a good performance in reducing the average AoI. Sometimes, however, to collect data from more clusters early on to lower the average, some data were picked up late.

**Figure 16:**
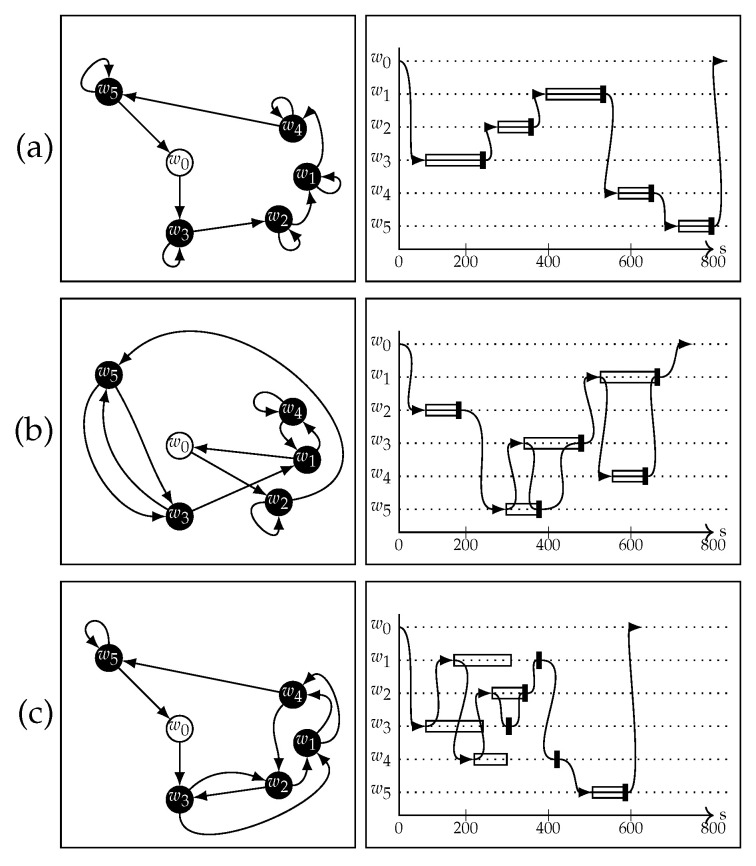
Example comparison of paths found by different strategies. Single round with wait (**a**), average AoI minimization (**b**), and average data-collection time minimization (**c**). Both Single Round with Wait and AoI minimization achieved zero AoI, as the data retrievals were scheduled for as soon as the computations were over. The second strategy, however, had a shorter overall mission time, as the UAV was not forced to remain idle when the clusters were busy with computation. The average data collection minimization scheduled more collections early on in the process and, without the constraint of having to pick up data as soon as possible, achieved a shorter mission time.

Minimizing the AoI was successful but led to overall poor performances for the other metrics. Nevertheless, the total wait time was still up to 39.13% better than the Single Round with Wait strategy. Removing the constraint of hovering in place until data pickup allowed for the computation to begin on other clusters before returning to retrieve the computation results. Figure 16a,b show the difference in performance between the Single Round with Wait strategy and the $BA_{2n+1}$ + double round used to minimize the average AoI on a specific instance of test configurations with five clusters. Figure 16c shows the path found by minimizing the average data collection time. Despite not picking up some data immediately, this strategy managed to schedule most data retrieval operations early on in the process.

## 5. Conclusions

In this work, we discussed a scenario of UAV-enabled data collection for WSN that had been poorly investigated in the literature. In this case, a UAV plays a very active role in the network as it is able to command concurrent computations in different clusters of WSN nodes and then collect the outcomes efficiently. This ability could open up a wide range of distributed applications in which a UAV is not only a mere collector of data, but can act on the network and reprogram it when needed. For instance, this possibility can be highly valuable in emergency scenarios to reprogram the network ad hoc, after complex maintenance operations to verify which nodes are properly functioning, and to prolong the lifespan of the network by making it perform demanding computations only when needed.

In our proposed solution to this problem computation times of clusters may overlap to allow for a UAV to move from one CH to another, resulting in overall shorter collection times with respect to the prevalent path optimization techniques adapted to the purpose. The simulation results show that approaches that do not exploit this characteristic of the studied problem achieve worse performances. The proposed solution can also be easily adapted to optimize different metrics, such as Average AoI, Average cluster computation end time, and Average data collection time, depending on the requirements of the use case, achieving satisfactory performances.

Future works will consider extending mission planning to multiple cooperating UAVs, using a variable number of visits per cluster, adjusting the mission plan to manage contingencies, and joint optimization of multiple metrics.

## Figures and Tables

**Table 1:** Definition of the additional metrics used for the optimizations described in Section 4.2.

Metric	Definition
Average AoI	$\frac{1}{\left|W\right|}\sum_{i=1}^{\left|W\right|}\left(t_{i^{\prime }}-t_{i}-\tau_{i}\right)$
Average cluster computation end time	$\frac{1}{\left|W\right|}\sum_{i=1}^{\left|W\right|}\left(t_{i}+\tau_{i}\right)$
Average data collection time	$\frac{1}{\left|W\right|}\sum_{i=1}^{\left|W\right|}t_{i^{\prime }}$

## Data Availability

Not applicable.
